# Infection and neonatal encephalopathy

**DOI:** 10.1038/s41390-025-04129-3

**Published:** 2025-07-09

**Authors:** Eleanor J. Molloy, Aoife Branagan, Cynthia Bearer, Eric S. Peeples

**Affiliations:** 1 Discipline of Paediatrics, Trinity College Dublin, The University of Dublin, Dublin, Ireland; 2 Trinity Translational Medicine Institute (TTMI), St James Hospital, Dublin, Ireland; 3 Trinity Research in Childhood Centre (TRiCC), Dublin, Ireland; 4 Neonatology, Children’s Health Ireland (CHI) at Crumlin & Tallaght, Dublin, Ireland; 5 Neurodisability, Children’s Health Ireland (CHI) at Crumlin & Tallaght, Dublin, Ireland; 6 Paediatrics, Coombe Women’s and Infant’s University Hospital, Dublin, Ireland; 7 Department of Pediatrics, Case Western Reserve University School of Medicine, Cleveland, OH USA; 8 Division of Neonatology, Department of Pediatrics, UH Rainbow Babies & Children’s Hospital, Cleveland, OH USA; 9 Department of Pediatrics, University of Nebraska Medical Center, Omaha, NE USA; 10 Division of Neonatology, Children’s Nebraska, Omaha, NE USA; 11 Child Health Research Institute, Omaha, NE USA

Neonatal encephalopathy (NE) is described as a ‘clinically defined syndrome of disturbed neurological function in the earliest days of life in the infant born at or beyond 35 weeks gestation manifested by subnormal level of consciousness or seizures, and often accompanied by difficulty with initiating and maintaining respiration and depression of tone and reflexes’ by the American College of Obstetricians and Gynecologists and the American Academy of Pediatrics.^1^ NE ranks globally in the top 10 neurological conditions with the highest age-standardised disability-adjusted life years (DALYs) and is increasingly recognised as a major cause of mortality and lifelong morbidity.^2^ The incidence of early culture-positive infections is much higher in the NE population, increased 20–40 fold over newborns without NE.^3^ Rao et al. reported significantly higher incidence of early confirmed infection in NE (23/1000) compared to rates of early onset sepsis in term and near term infants (0.5–1.0/1000 live births).^3^ Although the link between NE outcomes and infection, including chorioamnionitis and other maternal infections, has been well documented^4^ and there have been suggestions that therapeutic hypothermia (TH) is less effective in infants with sepsis and NE,^5,6^ this evidence has not yet altered TH guidelines.

Even in the absence of NE, early infection can significantly alter neonatal outcomes. Grether et al.^7^ demonstrated that chorioamnionitis was associated with brain injury and worse neurodevelopmental outcomes including cerebral palsy. This was further corroborated by Wu et al.^8^ who reported that chorioamnionitis was an independent risk factor for cerebral palsy from a large population-based cohort with case control design. Jenster et al.^9^ found a lower risk of moderate-severe brain injury and adverse childhood cognitive outcomes in children with NE with no evidence of chorioamnionitis. Additionally, watershed predominant brain injury was seen in children with NE who had signs of neonatal sepsis. However, in a cohort of 500 infants with NE, chorioamnionitis (clinical or histological) was associated with less sentinel events, less central pattern injury on MRI, fewer EEG background anomalies and lower severity of 2 year neurodevelopmental outcomes than unexposed infants.^10^ Congenital infections and viruses are also associated with significant neurological and neurodevelopmental issues.^11^

Using the National Neonatal Research database of 564,898 infants admitted in England and Wales over a 10-year period, Odd et al.^12^ studied risk factors for perinatal infection (at least one of the following: >18 h rupture of membranes, Group B *Streptococcus* colonisation, clinically diagnosed chorioamnionitis, maternal pyrexia, or antepartum antibiotics) and outcomes in infants with NE. They found that 998 (13.7%) of 7265 infants with NE had perinatal risk factors for infection. The primary outcomes of death and or nasogastric feeds were not different between infants with NE with or without exposure to these risk factors. However, infants exposed to perinatal risk factors for infection had decreased duration and number of antiseizure medications, as well as time to full sucking feeds. Odd et al.’s paper lacked data on the duration of antibiotic treatment, lumbar puncture results, magnetic resonance imaging (MRI) findings, placental pathology, early inflammatory markers, maternal infection, and antibiotic duration as well as longer term outcomes. Despite these limitations, their study supports the use of TH in infants at high risk of infection. Hakobyan also showed that culture positive sepsis was not a barrier to improved outcomes from TH in NE.^13^

In the hypothermia for moderate or severe neonatal encephalopathy in low-income and middle-income countries (HELIX) study, the combined outcome of death or disability at 18 months was not decreased by TH after neonatal encephalopathy in low-income and middle-income countries, and death alone was significantly increased.^14^ The HELIX study highlighted the frequency of perinatal and neonatal infection in NE, especially in low- and middle-income countries, and the need for further understanding of this aetiology and targeted treatments for these infants. Additional differences in the clinical phenotypes seen in these infants in south Asia versus those who were recruited into the original high-income country TH trials included lower intrapartum sentinel events, lower birth weights, increased white matter injury on MRI, earlier seizures and less severe acidosis, as well as different host genome expressions between the populations.^15,16^ Tann et al. also demonstrated increased bloodstream bacterial products on PCR in babies in Uganda versus controls, using a novel panel of real-time PCR assays.^17^

Neonatal sepsis has been associated with a watershed pattern of injury and white matter damage after NE.^18^ To better understand this interaction, some animal models of hypoxia-ischaemia have included LPS sensitisation^19–23^ to more closely approximate neonatal brain injury and outcomes in the population of infants with NE complicated by early infection. Although these LPS-preconditioned models have been described, they vary by pathogenic organism, with more injury gram negative versus gram positive organisms.^24^ In addition, the timing of the infectious insult alters outcomes as increased vulnerability of developing brain was found with early acute or days after LPS administration, whereas an intermediate interval between LPS and HI had the opposite effect.^24^ Ongoing research aims to match animal models with each major aetiology of NE and assess adjunctive therapies in addition to TH.

## Inflammation and infection in NE

Systemic inflammation and multiorgan dysfunction are common in NE and brain injury itself is associated with an increased risk of infection. For example, in traumatic brain injury and stroke there is an alteration in systemic immune function with increased risk of sepsis and pneumonia in both human and animal models.^25,26^ Following hypoxia-ischaemia, there may be alteration of protective mucosal barriers and bacterial translocation may occur. Immune dysfunction in NE is well-described and alters susceptibility to infection.^27–29^ Immune markers of inflammation such as C-reactive protein (CRP) and leucocytosis demonstrate a delayed elevation following NE and serial samples reveal a delayed peak.^30,31^ Negative blood culture results may not reliably eliminate early onset sepsis if maternal antibiotics have been administered antenatally as seen from the example of group B Streptococcus prophylaxis.^32^ In addition blood cultures are usually taken in the first hour of life when the infant may have only had direct bacterial exposure intrapartum and may not have enough bacterial growth to result in a positive culture and/or a limited blood volume available for culture. Recommendations from the Clinical and Laboratory Standards Institute consensus in 2022 noted that in adults, 60 ml of blood is considered sufficient for cultures to achieve >95% sensitivity, as over 50% of patients have bacteremia with only 1 colony forming unit.^33,34^ Therefore in neonates with blood culture samples of 1–2 mLs blood detection of microorganisms may be insufficient. In 1996 Schelonka et al. examined 95 neonatal blood cultures in vitro and inoculated them with common organisms. Since neonates are at risk of sepsis with a colony count less than 4 CFU/ml, a 0.5 ml inoculum of blood into the culture media was considered inadequate for sensitive and timely detection of bacteremia.^25^ The authors suggested 1–2 mLs of blood should increase microorganism detection if there is a low-colony-count sepsis but complete validation has not been performed,^25^ Although molecular tools for the detection of microorganisms, with pathogen-specific or broad-range PCR assays may be helpful, there are problems in the detection of DNA rather than a living organism, risk of contamination and no gold standard.^26^ Therefore, clinical details of maternal infection, antibiotic exposure, culture results and inflammatory markers are useful to assess the potential risk of infection in the infant.^32^

## Therapeutic implications of infection in NE

What can we do clinically today? Perinatal factors such as maternal pyrexia, fetal tachycardia and prolonged rupture of membranes, as well as other risk factors for sepsis should be rapidly reported. Development of mechanisms to ensure the neonatology team is updated rapidly on maternal infection including inflammatory markers, microbial cultures and sensitivities, especially in the age of drug-resistant organisms is vital to ensure early and appropriate treatment. Partially treated infection is a possibility in this scenario and so a complete septic workup may be indicated for ongoing or new-onset clinical signs such as apnoea and oxygen requirement.

Duration of antibiotics may need to be individualised. Lumbar puncture may be considered although it may be delayed until rewarming as it is challenging to perform during TH. Cautious use of antibiotics such as gentamicin are indicated due to frequent renal and hepatic insufficiency in NE.^35^ There is wide variation in antibiotic use internationally for infants with NE but as a group they have significantly greater rates of infection with increased severity of illness and the risk-based initiation of antibiotics is supported by the AAP guidance with scope for enhanced antimicrobial stewardship.^36,37^ Placental pathology is also vital to assess the degree of chorioamnionitis and its implications for neonatal management and longer-term outcomes and to update families on the aetiology. Early CRP rise has been correlated with histological chorioamnionitis in preterm infants and may have potential for future early classification of patient subgroups.^38^

## Future directions

Early recognition of potential sepsis and aligning with maternal markers of infection may aid appropriate antibiotic use and duration in NE. Serial monitoring of the baby for sepsis or infection and careful antimicrobial stewardship to standardise antibiotic use with ongoing quality improvement programmes may optimise antimicrobial management. Serial blood biomarkers of sepsis may be enlightening, especially in the context of TH which is associated with the delays in the rise of CRP. Placental pathology is helpful to provide aetiology and ongoing studies are assessing the potential associations between placental pathology and therapeutic outcomes. In animals, LPS sensitisation decreases the effectiveness of TH, therefore early recognition of chorioamnionitis will allow further developments of novel targeted therapies for this group of infants. The question of whether infection alters the effectiveness of TH in humans, however, seems to be more complex and likely depends on the timing and severity of exposure. Azithromycin and other older repurposed medications are under investigation.^3^ The absence of a consensus definition for either NE or neonatal sepsis, and neonatal organ dysfunction hampers aetiological classification and future directed therapies. Advanced methods for optimising antimicrobial use and detection of infection to develop novel therapeutics are on the horizon.^39,40^
